# Interstitial 7q31.1 copy number variations disrupting *IMMP2L* gene are associated with a wide spectrum of neurodevelopmental disorders

**DOI:** 10.1186/s13039-014-0054-y

**Published:** 2014-08-13

**Authors:** Stefania Gimelli, Valeria Capra, Maja Di Rocco, Massimiliano Leoni, Marisol Mirabelli-Badenier, Maria Cristina Schiaffino, Patrizia Fiorio, Cristina Cuoco, Giorgio Gimelli, Elisa Tassano

**Affiliations:** 1 Service of Genetic Medicine, University Hospitals of Geneva, Geneva, Switzerland; 2 U.O. Neurochirurgia, Istituto G.Gaslini, Genoa, Italy; 3 USD Malattie Rare, Istituto G Gaslini, Genoa, Italy; 4 Pediatria II, Istituto G Gaslini, Genoa, Italy; 5 DINOMGI Dipartimento-Università di Genova; U.O. Neuropsichiatria infantile, Istituto G. Gaslini, Genoa, Italy; 6 Dipartimento di Pediatria, Istituto G Gaslini, Genoa, Italy; 7 Laboratorio di Citogenetica, Istituto G. Gaslini, G.Gaslini 5, Genoa, 16147, Italy

**Keywords:** IMMP2L, Neurodevelopmental disorders, Copy number variation, Array-CGH

## Abstract

**Background:**

Since the introduction of the array-CGH technique in the diagnostic workup of mental retardation, new recurrent copy number variations and novel microdeletion/microduplication syndromes were identified. These findings suggest that some genomic disorders have high penetrance but a wide range of phenotypic severity.

**Results:**

We present the clinical and molecular description of four unrelated patients affected by neurodevelopmental disorders and overlapping 7q31.1 microdeletion/microduplication, identified by array-CGH and involving only part of the *IMMP2L* gene.

**Conclusion:**

*IMMP2L* encodes an inner mitochondrial membrane protease-like protein, which is required for processing of cytochromes inside mitochondria. Numerous studies reported that this gene is implicated in behavioural disorders such as autistic spectrum disorders, attention-deficit hyperactivity disorders, and Gilles de la Tourette syndrome. We discuss the functions of the gene suggesting that IMMP2L may act as risk factor for neurological disease.

## Background

The *IMMP2L* gene encodes the apparent human homologue of yeast mitochondrial inner membrane peptidase subunit 2 (MIM 605977). The human *IMMP2L* gene encodes the IMP2 protein and spans about 860Kb of genomic DNA. Yeast IMP2 is required for processing of i-cytochrome c1 and for stable expression of IMP1, which, in turn, is the protease required for processing of pre-COXII (cytochrome c oxidase subunit II) and i-cytochrome b2. Interestingly, mitochondrial proteins, including COXII, have been found associated with the appearance of neurodegenerative disorders [1].

Recently, genome-wide linkage studies have identified genomic imbalances involving *IMMP2L* gene in 7q31.1 that are associated with autistim spectrum disorders (AUTS9) (MIM 611015) [2], attention deficit/hyperactivity disorder (ADHD) (MIM 143465) [3] and Gilles de la Tourette syndrome (GTS) (MIM 137580) [4]–[7].

Autism spectrum disorders (ASD) are a subset of complex neurodevelopmental disorders characterized by reduced reciprocal social interaction, impaired ability to communicate, and a narrow range of interests and repetitive behaviours. ASD is clinically heterogeneous and is often associated with other conditions such as epilepsy and mental retardation [8],[9]. ADHD is a common and highly heritable disorder, but specific genetic risk factors remain elusive. GTS is a neurobehavioral disorder characterized by motor and vocal tics and behavioural abnormalities usually appearing between 3 and 8 years of age.

Here, we present the clinical and molecular data of four unrelated patients with overlapping 7q31.1 deletions/duplication disrupting *IMMP2L* and we discuss the functions of the gene in this region as well as the effects of copy number variations (CNVs) on the patients’ clinical features.

### Case presentation

Clinical data and array-CGH results are summarized in Table 1.

**Table 1:** **Clinical and molecular features of the four patients with interstitial 7q31.1 copy number variations disrupting** ***IMMP2L*** **gene**

	**Patient 1**	**Patient 2**	**Patient 3**	**Patient 4**
*IMMP2L* exons (n)	Ex3	Ex1, Ex2, Ex3	Ex1, Ex2, Ex3	Ex6
Gain/Loss	Loss	Loss	Loss	Gain
Size	269.6 kb	152.7 kb	249.9 kb	370.7 kb
Inheritance	Paternal	Paternal	Paternal	Paternal
Sex	F	M	M	M
Age at report	14 months	5	9	17 months
Birth weight (g)	3570	2830	n.a.	3420
Birth length (cm)	50	48	n.a	48
Birth OFC (cm)	34.5	35	n.a.	31
Weight (kg)	9.5 kg (25^th^)	19.6 kg (75^th^)	40.1 Kg (97^th^)	9.3 (<5^th^)
Height (cm)	78 cm (50^th^)	102 cm (3^rd^)	136.34 (75^th^)	78.5 (20^th^),
OFC (cm)	45.5 cm (25^th^)	52 cm (25-50^th^)		46.7 (25^th^)
Brain/CNS malformations	-	-	-	-
Psichomotor delay	-	+	+	+
Language delay	-	+	+	+
Behaviour problems	-	H*etero*-*aggressive, hyperphagia*	-	
Epilepsy	-	+	+	-
Hypotonic	-	Paratonia	+	+
Autism	-	Some autistic symptoms	-	-
Skull abnormalities	-	-	-	-
Skeletal	-	Brachydactily, flat feet	Scoliosis	-
Other malformations	Nystagmus	-	-	-
Other dysmorphic features	Mild facial dysmorphism, frontal bossing	Minor facial, *small hands* and *feet*	Arched palate, large central incisors	Frontal bossing, hypertelorism, saddle back nose, inverted buccal fissure, modest micrognathia
Others	Negative FRMD7 gene analysis	-	-	Negative MID1 gene analysis

#### Patient 1

The girl is the second child of unrelated parents. The mother shows autoimmune arthritis and hypothyroidism treated by levothyroxine also during pregnancy while the father is apparently healthy. Her brother at birth presented clubfoot and sacral dermal sinus. The mother reported a paternal aunt with breast tumour. The pregnancy was complicated by gestational diabetes and hypertension appearing at the eighth month of pregnancy. The patient was born at term by caesarean section. Apgar score was 9 at 1^st^ and 10 at 5^th^ minute, birth weight was 3570 g, head circumference (OFC) 34,5 cm, length 50 cm. The psychomotor development was normal until the age of 13 months. She was referred to the Istituto G. Gaslini because of horizontal and vertical nystagmus. Neurological evaluation confirmed the presence of nystagmus with inconstant eye contact and head deviation. Ophthalmological investigation did not identify any alteration of visual acuity. Brain MRI was normal. Abdominal ultrasound was unremarkable.

Mild facial dysmorphisms including frontal bossing, small head circumference (45.5 cm, 10^th^ centile), and deep-set eyes were present.

#### Patient 2

The proband is the only child of non-consanguineous healthy parents. No family history of neuropsychiatric disorders was reported. The child was born at 42 weeks of gestation by caesarean section after an uneventful pregnancy. At birth, weight was 2830 g, length 48 cm, and head circumference (OFC) 35 cm. Apgar score was 10 at 1^st^ minute.

After a normal perinatal period, his psychomotor development was evaluated as delayed: he was able to sit without support at 12 months and to walk at 18 months, and he did not develop any verbal language. He showed generalized seizures at 12 months of age that were treated with valproic acid. Brain MRI performed at the age of 16 months was normal.

At 5 years of age, the patient was admitted to Istituto Giannina Gaslini. On physical examination, body weight was 19.6 kg (75^th^ percentile), height 102 cm (3^rd^ percentile), BMI 18,8 (97^th^ percentile), and head circumference 52 cm (25-50^th^ percentile).

He showed minor facial dysmorphic features, small hands and feet, bilateral retractile testes. The estimated bone age corresponded approximately to 4 years-of age, with dissociation between the maturation of carpal bones and distal brachydactily.

Neurological examination showed paratonia, motor clumsiness, wide base gait, and flat feet. Furthermore, he presented oro-facial dyspraxia with open mouth posture, tongue protrusion and sialorrhea, and severe language impairment with inability to use any word with meaning.

The Griffiths Mental Development Scale, administered at 5 years of chronological age, demonstrated a global mental age of 2 years, with impairment of language as measured by a specific sub-scale.

Although the child demonstrated some autistic symptoms, as well as motor stereotypes and reduced sensitivity to pain, non-verbal communication skills such as pointing and joint attention, the DSM-IV diagnostic criteria for Autism Spectrum Diagnosis were not fulfilled. Moreover, he showed hyperactivity and attention deficit, hyperphagia, hetero-aggressive behaviours, and sleep disorders with frequent awakenings during the night.

Non-epileptic seizures occurred and the electroencephalogram was normal, without epileptiform discharge. Laboratory investigations showed IgA deficiency and subclinical hypothyroidism.

#### Patient 3

The patient is the first male child of healthy non-consanguineous parents born at term after a normal pregnancy. He has a family history of epilepsy. He was referred for developmental delay to the Istituto G.Gaslini at the age of 9 years. His weight was 40.1 Kg (97^th^ centile) and height 136.34 cm (75^th^ centile). His medical history included language and developmental delay with independent walking at 18 months, and idiopathic epilepsy until 3 years of age. An IQ of 75 was identified as borderline. He was affected by celiac disease and subclinical hypothyroidism. On physical examination, he presented scattered hypochromic spots, scoliosis, hypotonia with associated mild facial dysmorphisms including high arched palate and large central incisors.

#### Patient 4

A 17-month old boy was admitted to the Istituto G.Gaslini because of dysmorphisms and neurological problems. Family history included paternal grandfather with delayed psychomotor development which normalized at school age, and a maternal cousin of his father with delayed speech. He was born at term to consanguineous parents after a pregnancy characterized by threatened abortion at 3 months. At birth, weight was 3420 g, length 48 cm, head circumference (OFC) 31 cm, and Apgar score 9 at 1^st^ and 10 at 5^th^ minute.

In the first months of life, delay in the acquisition of psychomotor developmental milestones became progressively evident, with head control at 10 months, and babbling at 7–8 months. At the age of 17 months, his weight was 9.3 kg (<5^th^ percentile), height 78.5 cm (20^th^ centile), head circumference 46.7 cm (25^th^ centile), chest circumference 47.5 cm. He showed facial dysmorphisms: frontal bossing, hypertelorism, saddle back nose, inverted V rima oris, and modest micrognathia. He had hypotonia and global psychomotor development and language delay. He was not able to sit with or without support, he had not gained control of the trunk, and he did not pronounce meaningful phonemes. Neurological examination was normal. Severe constipation was reported, and therefore molecular analysis of *MID1* gene (MIDLINE1, MIM 300552) for Opitz G/BBB syndrome was performed. Sequence analysis of the gene was not able to identify any mutations and MLPA did not reveal any duplications or deletions (the analysis of the gene was performed in another hospital. Data on methods are not available).

## Results

### Patient 1

**Figure 1:**
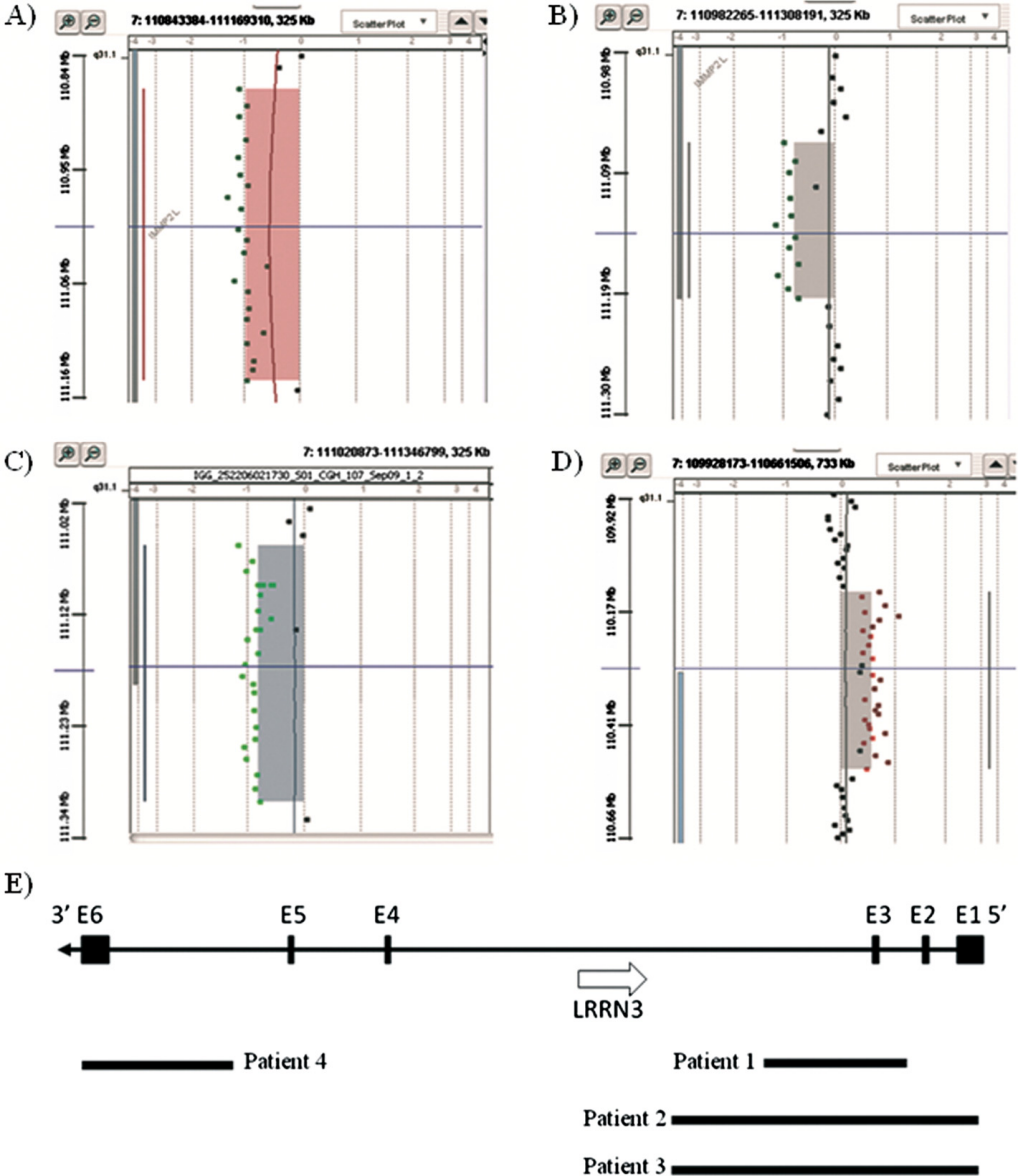
**Results of array-CGH analysis in our cases 1, 2, 3, and 4. A)** Array-CGH analysis in patient 1 shows ~ 269 kb deletion at 7q31.1 (chr7:110,879,586-111,149,166) including exon 3 of *IMMP2L* gene. **B)** Array-CGH analysis in patient 2 shows a ~ 152.7 kb deletion at 7q31.1 (chr7:111,066,736-111,201,968). The deletion encompasses exons 1, 2, and 3 of IMMP2L gene. **C)** Array-CGH analysis in patient 3 shows the presence of ~249.9 kb deletion at 7q31.1 (chr7:111,066,736-111,316,651) encompassing exons 1, 2, and 3 of IMMP2L gene. **D)** Array-CGH analysis in patient 4 shows a ~307.7 kb duplication at 7q31.1 (chr7:110,135,083-110,505,806) including exon 6 of *IMMP2L* gene. **E)** Schematic representation of deleted regions in patients 1, 2, and 3 and of duplicated region in patient 4.

Array-CGH analysis revealed the presence of a ~ 270 kb deletion at 7q31.1, arr [hg19] 7 q31.1(110,879,586-111,149,166)x1, including the exon 3 of *IMMP2L* gene (Figure 1A). The deletion was inherited from her healthy father.

Further sequence analysis of *FRMD7* gene (MIM 300628) within the critical interval for X-linked congenital nystagmus (NYS1; MIM 310700) on Xq26-q27 was normal and MLPA screening did not reveal any deletions or duplications within the region.

### Patient 2

Karyotype analysis was normal, while array-CGH demonstrated the presence of an ~ 153 kb deletion at 7q31.1, arr [hg19] 7q31.1(111,066,736-111,201,968)x1, encompassing exons 1, 2, and 3 of *IMMP2L* gene (Figure 1B). The deletion was inherited from his healthy father.

### Patient 3

He had a normal karyotype and array-CGH analysis showed the presence of ~250 kb deletion at 7q31.1, arr [hg19] 7q31.1(111,066,736-111,316,651)x1, encompassing exons 1, 2, and 3 of *IMMP2L* gene (Figure 1C). The deletion was inherited from his healthy father.

### Patient 4

Array-CGH revealed the presence of a first ~171 kb duplication at 7p22.1, arr [hg19] 7p22.1(4,785,596-4,956,419)x3, including five genes (*FOXK1, AP5Z1, RADIL, PAPOLB, MMD2*) and a second ~308 kb duplication at 7q31.1, arr [hg19] 7q31.1(110,135,083-110,505,806)x3, including exon 6 of *IMMP2L* gene (Figure 1D). Array-CGH of the parents showed that the child inherited the 7p22.1 duplication from his mother and the 7q31.1 duplication from his father.

## Discussion

Thanks to the widespread use of array-CGH in the diagnostic workup of mental retardation (MR), new recurrent CNVs and novel microdeletion/microduplication syndromes have been described. The introduction of array-CGH led to the identification of an increasing number of individuals carrying CNVs. These findings highlighted the presence of a strong connection between genomic disorders with high penetrance and a wide range of phenotypic severity. The recurrent CNVs identified in multiple unrelated patients were often associated with a broader range of phenotypes.

A recent *AUTS1* fine-mapping study, using both family-based and case–control association analyses, detected SNPs within *DOCK4* (dedicator of cytokinesis 4) and *IMMP2L* (IMP2 inner mitochondrial membrane protease-like) that may be indexing autism susceptibility factors [2].

Incomplete processing of IMMP2L substrates seems to result in a hyperactive mitochondrion with an increased production of superoxide [10] and to promote the release of pro-apoptotic proteins, thereby activating cell death pathways [11],[12].

Interestingly, COXII and other mitochondrial proteins have been found associated with the appearance of neurodegenerative disorders, and stroke-like episodes [13],[14], and they have been hypothesized in neuropsychiatric disorders [15]. Moreover, *IMMP2L* contains a neuronal leucine-rich repeat gene (*LRRN3*), highly expressed in fetal brain, nested within its large third intron. Murine and Drosophila studies demonstrated that many members of the LRR family play an essential role in target recognition, axonal path finding, and cell differentiation during neuronal development [16],[17].

*IMMP2L* gene has been indicated as a possible candidate for Gilles de la Tourette syndrome [4]–[7].

Here we report on three cases with microdeletions and one with a microduplication, detected by array-CGH encompassing a region that includes only a portion of *IMMP2L.* The copy number variation size ranges from ~150 Kb to ~370 Kb. The microdeletions identified in patients 2 and 3 affect exclusively the first three exons, the deletion in patient 1 affects only exon 3, and the microduplication observed in patient 4 includes exclusively exon 6. Additional 7p22.1 duplication present in our patient 4 was inherited from his mother. To our knowledge this duplication contains five genes, i.e. *FOXK1, AP5Z1, RADIL, PAPOLB, MMD2* that till now, could not seems to be related with a neurologic phenotype. No additional CNVs were identified in the other patients (Table 2).

**Table 2:** Summary of the microdeletions/microduplications

**Patient**	**Sex**	**Deletion duplication**	**Rearrangement size (Kb)**	**Exons involved**	**Parental origin**	**Parental phenotype**	**Additional CNVs**
Patient 1	F	Chr7:110.879.166_111.149.166 del	270	3	Paternal	Unaffected	
Patient 2	M	Chr7:111.066.736_111.201.968 del	153	1, 2, 3	Paternal	Unaffected	
Patient 3	M	Chr7:111.066.736_111.316.651 del	250	1, 2, 3	Paternal	Unaffected	
Patient 4	M	Chr7:110.135.083_110.505.806 dup	308	6	Paternal	Unaffected	Chr7:4.785.596_4.956.419 dup 171 Kb, maternal duplication which includes *FOXK1*, *AP5Z1*, *RADIL*, *PAPOLB*, *MMD2*

Recently, Bertelsen et al. [7], through screening of a Danish cohort comprising 188 unrelated Tourette syndrome patients, reported seven patients with intragenic *IMMP2L* deletion suggesting it as a susceptibility factor for Tourette syndrome. These authors reported that the deletion frequency was significantly higher in their cohort compared with the background population and the Affimetrix reference cohort.

Generally, GTS syndrome appears at 2 to 14 years of age. None of our patients showed signs of the GTS syndrome, but only patients 2 and 3 were the age at which symptoms of this syndrome may occur, the other two were much younger and could develop these symptoms in the future.

However, it is likely that *IMMP2L* deletions are not fully penetrant, as underlined by the finding of deletions/duplications in unaffected fathers (Table 1) and in control populations reported in the Database of Genomic Variants. Seven cases of deletions and one of duplication overlapping the 7q31.1 region including only *IMMP2L* have been reported in the DECIPHER database (275553, 263106, 257170, 262064, 271698, 283475, 282171, 280009, 288959) but only three deletions and the duplication have a phenotype description. The patients with 7q31.1 deletion presented unspecified intellectual disability and behavioural/psychiatric abnormalities while the one with duplication showed abdominal situs inversus and dextrocardia.

All the patients described here showed dysmorphisms, three presented hypotonia, psichomotor and language delay, two had epilepsy, and three showed microcephaly. Only one patient showed some autistic symptoms, while two showed mild skeletal defects (Table 1).

Apparently, our patient 1 presented microcephaly, mild facial dysmorphisms, and nystagmus. No mutations were found in *FRMD7* gene (MIM 300628) involved in the pathogenesis of the nystagmus of the child. In our patient 4, *MID1* gene analysis, responsible of Opitz G/BBB syndrome, was negative.

Mitochondrial dysfunction was found associated with a range of human disorders, including neuropsychiatric disorders [18]. Therefore, defective IMMP2L may lead to apoptosis due to a hyperactive mitochondrion, as suggested by Bertelsen et al. [7], or may be a risk factor affecting myelination [19].

As perturbation of mitochondrial function has serious consequences in neurons, mutations associated with mitochondrial proteins are a highly likely to be associated with neurological diseases. Possible effects of the 7p22.1 duplication identified in our patient 4 remain unknown at the moment.

## Conclusions

In conclusion, our data suggest that partial deletions/duplications of *IMMP2L* gene may act as risk factors for neurological diseases and not only for GTS syndrome.

In our opinion, these data may add to the emerging theme that the same genomic variants, in combination with distinct genetic backgrounds, may contribute to different phenotypes.

Further studies on larger case series with *IMMP2L* CNVs are necessary to gain a better insight into the role of this gene in disease pathogenesis.

## Methods

Standard GTG banding was performed at a resolution of 400–550 bands on metaphase chromosomes from peripheral blood lymphocytes. Molecular karyotyping was performed on the probands and their parents using Human Genome CGH Microarray Kit G3 180 (Agilent Technologies, Palo Alto, USA) with ~13 Kb overall median probe spacing. Labelling and hybridization were performed following the protocols provided by the manufacturers. A graphical overview was obtained using the Agilent Genomic Workbench Lite Edition Software 6.5.0.18.

### Consent

Written informed consent was obtained from the parents of all patients for publication of this paper and any accompanying images. A copy of the written consent is available for review by the Editor-in-Chief of this journal.

## Competing interests

The authors declare that they have no competing interests.

## Authors’ contributions

All authors have made substantial contributions to conception and design, acquisition of data, analysis and interpretation of data. All authors have been involved in drafting the manuscript and revising it critically for important intellectual content. All authors read and approved the final manuscript.
